# Diagnosing a Strong-Fault Model by Conflict and Consistency

**DOI:** 10.3390/s18041016

**Published:** 2018-03-29

**Authors:** Wenfeng Zhang, Qi Zhao, Hongbo Zhao, Gan Zhou, Wenquan Feng

**Affiliations:** 1 Electronic and Information Engineering, Beihang University, Beijing 100191, China; zhangwenfeng42153@buaa.edu.cn (W.Z.); zhaoqi@buaa.edu.cn (Q.Z.); buaafwq@buaa.edu.cn (W.F.); 2 The 6th Research Institute of China Electronics Corporation, Beijing 100083, China; zhouganterry@163.com

**Keywords:** fault diagnosis, model-based diagnosis, truth maintenance system, conflict directed A*, a strong-fault model

## Abstract

The diagnosis method for a weak-fault model with only normal behaviors of each component has evolved over decades. However, many systems now demand a strong-fault models, the fault modes of which have specific behaviors as well. It is difficult to diagnose a strong-fault model due to its non-monotonicity. Currently, diagnosis methods usually employ conflicts to isolate possible fault and the process can be expedited when some observed output is consistent with the model’s prediction where the consistency indicates probably normal components. This paper solves the problem of efficiently diagnosing a strong-fault model by proposing a novel Logic-based Truth Maintenance System (LTMS) with two search approaches based on conflict and consistency. At the beginning, the original a strong-fault model is encoded by Boolean variables and converted into Conjunctive Normal Form (CNF). Then the proposed LTMS is employed to reason over CNF and find multiple minimal conflicts and maximal consistencies when there exists fault. The search approaches offer the best candidate efficiency based on the reasoning result until the diagnosis results are obtained. The completeness, coverage, correctness and complexity of the proposals are analyzed theoretically to show their strength and weakness. Finally, the proposed approaches are demonstrated by applying them to a real-world domain—the heat control unit of a spacecraft—where the proposed methods are significantly better than best first and conflict directly with A* search methods.

## 1. Introduction

As human beings rely on sophisticated systems more than ever, keeping their operation safe is quite significant for the environment, lives and property. Although the systems usually undergo rigorous tests before deployment, they may still be faulty because of wear, degradation and unexpected harm. Diagnosing their faults in time can stop the faults from spreading to other parts, resulting in irretrievable disaster [1,2,3].

Numerous diagnosis methods have been proposed in the last few decades [4,5,6,7,8] and most of them come from two domains: Artificial Intelligence (AI) [9] and Control Theory [3]. The methods can also be divided into two classes based on where system information comes from: the model-based method [10,11] and the data-driven method [12,13].

This paper only focuses on the Model-Based Diagnosis (MBD) proposed by Reiter [9] in the DX (Diagnosis from first principle) community, where MBD adopts reasoning methods in AI to diagnose by discrete model, MBD is composed of modeling (for example, first order logic) and reasoning. The reasoning can be further separated into two steps: conflict recognition and hitting sets generation. The hitting sets of all minimal conflicts have been proven to be the diagnoses for a weak-fault model theoretically [9]. Kleer and Williams [14,15] also independently found the theory which, however, focuses on getting all the conflicts by Assumption-based Truth Maintenance System (ATMS) [16,17]. The validity of the theory has been verified by the General Diagnosis Engine (GDE) [15,18]. But GDE consumes plenty of computation resources when diagnosing complex systems due to “combinatorial explosion.”

Practically speaking, only a few diagnosis solutions with a high weight are useful, which means not all the candidates should be analyzed. There are two types of famous approximation approaches to ignoring low weight candidates: a stochastic approach and a search approach.

The most famous stochastic approach is Safari [19,20] proposed by Feldman. Safari first assumes that all faults exist. Then it selects one fault randomly, sets it as normal (flip the state value) and obtains a new candidate. If the candidate causes conflicts, the flip is retreated and selects a fault randomly again. If there is no conflict, the random selection is applied to the new candidate. A diagnosis solution (the latest candidate) is obtained if there are conflicts in the last *n* (a preset parameter) random selection. Safari is optimal in probability for a weak-fault model and more efficient compared with search-based diagnosis methods. However, the optimality of Safari is not ensured for a strong-fault model [20]. So, this method is not chosen as the basis of this paper.

Search approaches usually obey the idea of “guess and verify”: they employ a search method to guess a most probable candidate first and then verify if the candidate is a valid diagnosis. Williams [18] and Kurien [21] proposed the Best-First (BF) diagnosis method, which utilized a search algorithm to find the candidate with maximal priori probability first and then checks whether there are conflicts between the system model, observation and the candidate. A diagnosis is obtained when a candidate with no conflict is found. Further, Williams proposed the Conflict Directed A* (CDA*) [22] search method which selected the best candidates based on a priori probability and verified them as well. But CDA* caches conflict sets recognized by the Logic-based Truth Maintenance System (LTMS) [23] and the verification for some candidates is skipped if they contain some conflicts. In this way, CDA* avoids much unnecessary computation and saves a lot of time. As one of the most important factors for diagnosis, conflict is widely studied. Stern explored the duality between conflict and diagnosis [24] and some divide and conquer methods [25,26,27] are proposed to reduce the cost to find conflicts.

The key techniques in the search approaches discussed above are how to utilize a priori information and conflict, which are also important research points in the DX community recently. However, much research concentrates too much on conflict to notice that even the consistency between model prediction and observation contains diagnosis information: the part in the candidate, which causes the model prediction, is probably right. Besides, traditional methods are proposed for a weak-fault model with only one normal mode and one unknown fault mode. The correctness is not ensured for a strong-fault [19] model in which fault modes are described with specific behaviors.

This paper studies conflict and consistency directed diagnosis methods for a strong-fault model comprehensively and has the following novelties: (1) A new conception, *consistency* (formally defined in Section 3.2.2), is proposed to boost diagnose; (2) a novel LTMS is proposed to obtain multiple conflicts and consistencies in one reasoning process; (3) proposes a more accurate distribution model about candidate and observation; (4) based on the distribution in (3), proposes two A* search-based algorithms that make full use of conflicts and consistencies to get single-diagnosis and multiple-diagnosis respectively.

The rest of this paper is organized as follows: Section 2 introduces the formal definitions and methods about system model, diagnosis problem and the conflict directed A* for a weak-fault model; Section 3 discusses the proposed method in detail; Section 4 analyzes the algorithms proposed comprehensively; Section 5 demonstrates the proposed methods by a real-word model: a heat control unit of a spacecraft; The last section presents the conclusion and future work.

## 2. Theoretical Background

This section introduces the basic definitions and methods about DX diagnosis methods.

### 2.1. Discrete Model

In DX, models are usually logic propositions in which variables are discrete: *Boolean* or *Enumerative*. Based on the description manner of discrete variables, the models are divided into weak-fault and a strong-fault models.

#### 2.1.1. Logic Based Diagnosis Model

Logic (such as first order logic) is a powerful language to describe complex behaviors and used in many famous diagnosis systems such as GDE and Sherlock [14].

Formally, a diagnosis model (DM) is defined by tuple (1).

<*SV*, *SD*, *MD*> (1)

where:

- *SV* (system variable) is the finite set of system variables. The set can be partitioned into mode variables (*MODE*), observation variables (*OBS*) and inner variables (*INNER*). The possible behavioral modes of each component are defined by the mode variable. Observation variables means their values can be obtained from sensors and in this paper (and other most cases) they are assumed to be perfectly correct. And inner variable is all other variables.
- *SD* (system description) is a finite set of logic propositions which are constraints over *SV*. Usually *SD* is represented by CNF or Negative Normal Form (NNF) [28].
- *MD* (mode distribution) is the prior probability distribution of mode variables.

Usually, models for components are first built and then are conjoined as (2) where *C* is a finite set of logic propositions, which describes how subsystems connect to each other.

(2) $$\begin{array}{l} SV=\cup SV_{i}\\ SD=(\cup SD_{i})\cup C\\ MD=\cup MD_{i} \end{array}$$

#### 2.1.2. Weak-Fault Model and Strong-Fault Model

The model which has only one normal mode and an unknown fault mode is called a weak-fault model. On the contrary, a model with specific fault modes is a strong-fault model. In other words, given the inputs, the outputs are determined for each fault mode in a strong-fault model.

(3) $$\begin{array}{l} SV=\{mode,in1,in2,out\},mode\in \{normal,fault\},in1,in2,out\in \{true,false\}\\ SD=\{(mode==normal)\Rightarrow (in1\;and\;in2==out)\}\\ MD=\{P(mode=normal)=0.99,P(mode=fault)=0.01\} \end{array}$$

**Figure 1:**
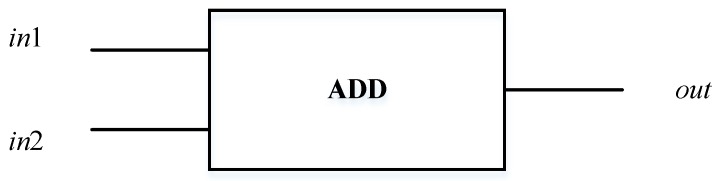
Adder.

The weak-fault model of the adder in Figure 1 is shown in (3). In this weak-fault model, the system has only two modes: *normal* and *fault*. When the mode is *normal*, *out* must be equal to “*in*1 *and in*2.” When the mode is *fault*, there is no constraint over inputs and output. Sometimes a weak-fault model uses a bool variable as the mode variable instead.

(4) $$\begin{array}{ll} SV= & \left\{mode,in1,in2,out\},mode\in \{normal,or\_fault\},in1,in2,out\in \{true,false\right\}\\ SD= & \{(mode==normal)\Rightarrow (in1\;and\;in2==out),\\ & \left(mode==or\_fault)\Rightarrow (in1\;or\;in2==out)\right\}\\ MD= & \left\{P(mode=normal)=0.99,P(mode=or\_fault)=0.01\right\} \end{array}$$

The strong-fault model of the adder in Figure 1 is shown in (4). Compared with model (3), the strong-fault model has a fault mode *or_fault*, in which *out* is equal to “*in*1 *or in*2” by mistake. The most significant difference between the two models is that the strong-fault model specifies the behaviors even for fault mode.

Diagnosing a strong-fault model is quite difficult because of the non-monotonicity. In a weak-fault model, setting a component as a fault will never cause a conflict because there is no constraint in fault mode. The famous Safari [20] utilizes this feature and starts diagnosis by assuming that all components are faulty. In a strong-fault model, if a mode’s behavior is not consistent with the observation, anyone in the rest may be available.

### 2.2. Diagnosis Problem

Given a DM, fault diagnosis is to find diagnosis solutions (diagnosis for short) that explain the health states of systems. This subsection gives some definitions formally. They are a little different from the definitions in other literatures [20,29] to be more suitable for a strong-fault model.

A Diagnosis Problem (DP) is defined by tuple (5).

<*DM*, *obs*> (5)

where:

- *DM* is the diagnosis model of a system.
- *obs* is the assignment set of observation variables.

#### 2.2.1. Diagnosis

A diagnosis *ω_s_* is an assignment set of mode variables that is consistent with the system model and the observation as (6). The diagnosis discussed in this paper is static.

(6) $$SD\cup obs\cup \omega_{s}|\neq ⊥$$

#### 2.2.2. Preferred Diagnosis

The definition of diagnosis above only describes what a diagnosis is but what is a good diagnosis is not given. Usually, diagnosis is evaluated by posterior probability *P*(*mode*|*obs*) which can be decomposed by Bayes rule as in Equation (7).

(7) $$P(mode|obs)=\frac{P(mode)P(obs|mode)}{P(obs)}$$

where:

- *P*(*mode*) is the priori probability of *mode*.
- *P*(*obs*|*mode*) is the conditional probability of observation *obs* given *mode*. For simplification, in most cases, *P*(*obs*|*mode*) is restricted in {0, 1} (when there exist conflicts, *P*(*obs*|*mode*) = 0; when there is no conflict, *P*(*obs*|*mode*) = 1) which means the diagnosis is evaluated by priori probability instead of posterior probability. However, the simplification ignores system structure and implies that the existence of conflict is the only flag of if the candidate *mode* is right, which means that both false alarm and missing alarm are zero. This paper proposes a new scheme to compute it more accurate.
- *P*(*obs*) is the marginal probability of observation *obs* and there is no need to compute it because it is a normalization factor.

Most approximation diagnosis methods [19] design a routine based on (7) to search for the best candidate and then check the consistency between the candidate and observation until the diagnosis (consistent candidate) is found. To the author’s knowledge, the top two famous methods are Best First A* (BFA*) search and Conflict Directed A* (CDA*) search. BFA* uses priori information (*P*(*mode*)) only and CDA* uses both priori (*P*(*mode*)) and conflicts information (parts of *P*(*obs*|*mode*)). As CDA* is the one of the state of art methods and the basis of our approaches, it is introduced briefly in the next subsection.

### 2.3. Conflict Directed A* Search for Weak Model

The search space size of a diagnosis problem is *d^n^*, where *d* is the domain’s average size of each mode variable and *n* is the size of set *MODE*. It is too costly to find all the diagnoses. However, most diagnoses are insignificant because of their low probabilities. CDA* employs the theorem about diagnosis and conflict to avoid testing unnecessary mode assignments.

A conflict *c* is an assignment set of mode variables which satisfies that

(8) SD∪obs∪c|=⊥

Without confusion, we also call the difference between observed value and predicted value a conflict (or between predicted values).

If *C* is a set of sets *c*_1_, *c*_2_ … *c_n_*, set *h* is a hitting set of *C* if

(9) ∀c∈C,h∩c≠∅

If *C* includes all the conflict sets, *c* is sure to be a diagnosis.

**Figure 2:**
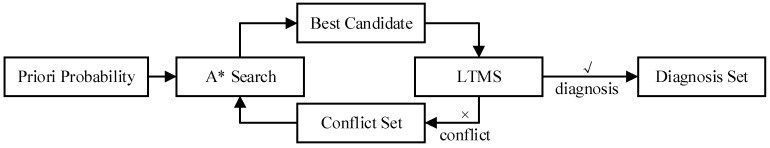
Framework of Conflict Directed A*.

To find the best candidate effectively, the conflict directed A* method develops a novel framework shown in Figure 2. The A* search finds a best candidate based on priori probability and the conflicts obtained till now. LTSM checks the consistence of the candidate, the system model and observation. If it is consistent, the candidate is a diagnosis and added into the diagnosis set. Otherwise, LTMS finds the minimal conflicts, adds them into conflict set and a new iteration starts. Williams introduces two distinct search algorithms for a single-diagnosis and multiple diagnoses.

#### 2.3.1. Single-Diagnosis

To find only one diagnosis, Williams generates a hitting set of current conflicts as the new candidate to be checked. To get the preferred hitting set efficiently, CDA* generates *constituent kernels* (CK) of each conflict first and then generates *kernels* that resolve all the conflicts.

A *constituent kernel* of conflict *c* is an individual assignment that resolves *c*.

A *kernel* of the set *C* including all conflict sets is the minimal set that resolves all conflicts in *C* (no subset of *kernel* resolves all conflicts).

**Figure 3:**
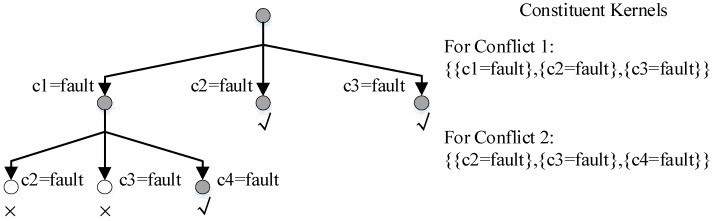
A Search Tree to Find *Kernels* of Conflict 1 and Conflict 2.

The A* search is employed to find a specific kernel. Figure 3 shows a search tree created by CDA* where blank nodes are invalid nodes and grey nodes are valid nodes. The two blank nodes are not kernels because they are not minimal and the two nodes in the right are not extended because they are able to resolve both conflicts.

When there is no conflict in the beginning, the candidate assumes that all components are *normal*. When conflicts are found by LTMS, the conflict manager generates the next best *kernel* by resolving all the conflicts and gets the next best candidate by setting all other components as *normal*. To be brief, Figure 3 evaluates candidates by the number of fault component and *kernels* are best candidates. In practice, *kernels* are also evaluated by probability.

#### 2.3.2. Multiple Diagnoses

**Figure 4:**
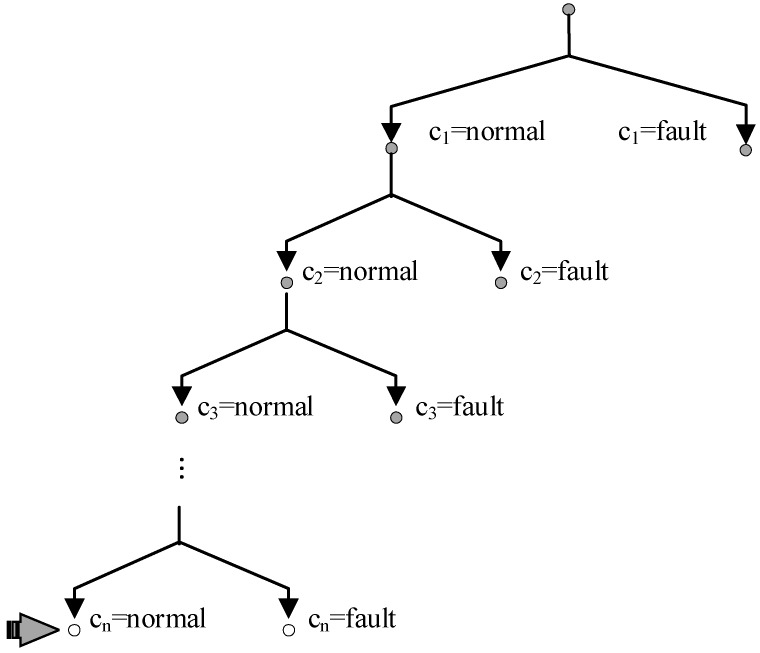
A Search Tree Expanded by Conflict Directed A* Search (CDA*) for Multiple Diagnoses.

When multiple diagnoses are expected, the diagnosis method is quite different. Figure 4 shows a search tree where blank nodes are candidates and grey nodes will be extended in the following search.

CDA* computes the cost of nodes in the search tree according to the prior probability. But if a node contains any conflict, its cost is ∞ and the node is skipped. A valid best candidate is found at the leaf node and it is a diagnosis if verified by LTMS. The search-verification iteration ends until enough diagnoses are obtained.

The next section will introduce the shortcomings of current methods and our solutions.

## 3. Conflict and Consistency Directed A* Diagnosis for a Strong-Fault Model

CDA* makes full use of conflicts that imply the scope of possible fault components. It is more effective than BFA* which will not jump over apparently possible candidates but there are still some drawbacks: (1) Although more conflicts can help to find best candidate more accurately, LTMS can only give one conflict one time; (2) Only conflict is utilized to obtain best candidate but *consistency* between predicted value and observed value implies the possible normal components; (3) The effectiveness of CDA* is not proven for a strong-fault model.

**Figure 5:**
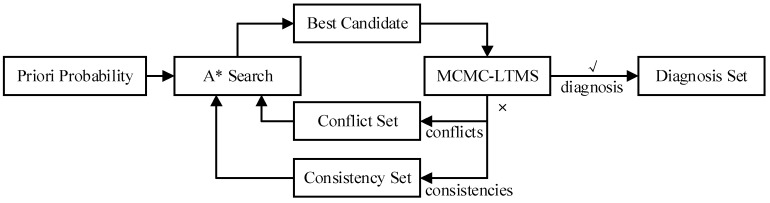
Framework of Conflict and Consistency Directed A* (CCDA).

To overcome the drawbacks above, this paper proposes a Conflict and Consistency Directed A* diagnosis method illustrated in Figure 5. The Multi-conflict and Multi-consistency LTMS (MCMC-LTMS) checks the consistency of candidate, model and observation and finds as many conflicts and consistencies as possible if it is inconsistent. The A* search generates the best candidate based on priori probability, conflicts and consistencies. The iteration continues until there are enough diagnoses in diagnosis set.

To accomplish this framework, three problems must be solved. The first one is to represent the model by Boolean variables so that classic reason technique such as unit propagation [30] can be applied. The second problem is MCMC-LTMS, which is quite difficult to achieve because there are insignificant consistencies generated in reasoning. They must be identified and ignored to avoid disturbing the generation of best candidate. How to utilize all the information to generate the best candidate is the last problem. This paper uses the encoding technique in Torta’s thesis [29] to address the first one and proposes innovative methods to solve the last two.

### 3.1. Model Encoding

Since there are multi-valued variables (enumeration variables) in a strong-fault model, LTMS cannot reason the system directly. Fortunately, multi-valued variables can be encoded by Boolean variables.

A multi-valued variable *v* (*v* = *v*_1_, *v*_2_ ... *v_n_*) with *n* values can be encoded by *n* Boolean variables where the value (*true*, *false*) of each Boolean variable indicates if the value of *v* is *v_i_*. Then the assignments in *SD* are replaced by Boolean variables as shown in (10) where *v* is a multi-valued variable, *v*@*v_i_* and *b_i_* are Boolean variables.

(10) $$\begin{array}{l} \mathrm{multi}-\mathrm{valued}\;\mathrm{variables}:v=v_{1},v_{2}\ldots v_{n}\\ \mathrm{boolean}\;\mathrm{variables}:v@v_{1},v@v_{2}\ldots v@v_{n}\\ \mathrm{origninal}\;SD:(v=v_{i})\wedge b_{1}\wedge !b_{2}\\ \mathrm{encoded}\;SD:v@v_{i}\wedge b_{1}\wedge !b_{2} \end{array}$$

Besides the replacements in *SD*, additional constraints are inserted to ensure that the assignments of Boolean variables are consistent with Enumeration variables. The first one is *MUTEXT* constraint, as (11), which ensures that any two Boolean variables encoding the same multi-valued variable will not be *true* at the same time.

(11) $$MUTEXT:!(v@v_{i}\wedge v@v_{j})$$

The second one is the *COMPLETENESS* constraint, as (12), which ensures that there must be at least one Boolean variable encoding multi-valued *v* is *true*.

(12) $$COMPLETENESS:v@v_{1}\vee v@v_{2}\vee \cdots \vee v@v_{n}$$

The two constraints make sure that there exists one and only one *true* variable in any set of Boolean variables encoding an Enumeration variable. By replacing the assignments and adding the extra constraints, original model is converted into a Boolean one that can be solved by LTMS.

### 3.2. Multi-Conflict Multi-Consistency LTMS

**Figure 6:**
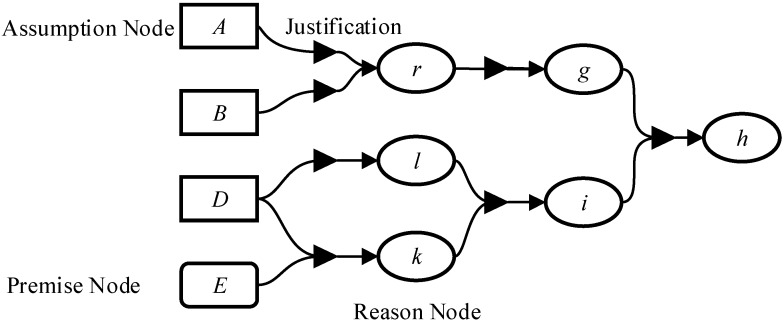
Logic based Truth Maintenance System (LTMS).

LTMS [23] is designed to avoid repeating reasoning. It is composed of node and justification as Figure 6. Nodes can be classified into assumption node, premise node and reason node. In diagnosis, premise nodes are observations and clauses of system model. Justification recording the reasoning process is mainly implemented by unit propagation [31]. All the reasoning rules are illustrated in (13) where (*a*) is a standard unit propagation which predicts a value of an inner variable/output variable, (*b*) is a true propagation which means the clause is *true* and (*c*) is a false propagation which means the clause is *false*. The consistency among candidate, model and observation is verified by the three rules.

(13) $$\begin{array}{l} (a)A=false,A\vee B\Rightarrow B=true\\ (b)A=false,B=true,A\vee B\Rightarrow true\;clause\\ (c)A=false,B=false,A\vee B\Rightarrow false\;clause \end{array}$$

When there is a conflict found in traditional LTMS, the reasoning is stopped. Then, the conflict sets are returned to obtain best candidate.

Although LTMS provides conflict sets to jump over many impossible candidates, it can only get one conflict every time and does not dig the information in system structure deeply. In this subsection, LTMS is improved in two perspectives: multiple conflicts and consistencies.

#### 3.2.1. Multi-Conflict LTMS

**Figure 7:**
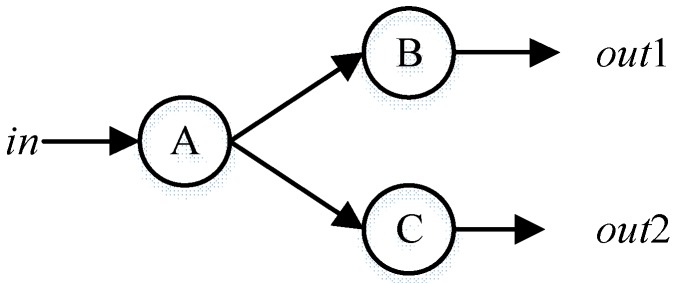
Structure of a Simple System.

More conflicts may help to greatly reduce the range of fault components. For example, in Figure 7, if the predicted value and observed value of *out*2 are different, we can get a conflict set {A = normal, C = normal} which indicates that there may be fault in component A or C but the better candidate can only be determined by priori probabilities. However, if another conflict, for example at *out*1, is found, it is obvious that component A is more likely to be faulty.

To get multiple conflicts in one reasoning process, multi-conflict LTMS does not stop when one conflict is found. To obtain as many conflicts as possible, this paper proposes an *out-ignorance in reasoning* strategy which means the observed output values do not participate in the reasoning. This technique makes a value can be propagated sequentially even if there is a conflict between predicted and observed value. This helps the reasoning to cover as many components as possible. Conflicts are found in two ways: (1) the differences between predicted values and observed values; (2) false clause as (*c*) in (13). Conflict sets are obtained by searching back forward until all the assumed modes causing conflicts (or fault clauses) are found.

#### 3.2.2. Multi-Consistency LTMS

Conflict reveals the scope of fault components. In the opposite, is there something indicating the range of normal component? Still in Figure 7, conflict {A = normal, C = normal} indicates both components A and C are possible to be faulty. But if there predicted value and observed value at *out*1 is the same, it implies that both component A and B are normal. So, component C is the one probably to be faulty. The example shows the basic idea of *consistency,* which is formally defined as follows:

A consistency *c* is an assignment set of mode variables satisfies that (1) there exits an over determined clause set [32] *SD_c_* in which any mode variable also exists in *c*; (2) there is no conflicts over *c*, *SD_c_* and observation *obs* as (14):

(14) $$c\cup SD_{c}\cup obs|\neq ⊥$$

A consistency *c* is *maximal* if there is no super set of *c* is a consistency. Different from multi-conflict LTMS, multi-consistency finds *maximal consistencies*.

Consistency is found by *true* clause but not all *true* clauses are *consistencies*. For example, (*in* == *out*) can be converted to ((!*in*∨*out*)∧(*in*∨!*out*)), where (*in*∨!*out*) is *true* when *in* is observed to be *true*. However, the *true* clause is insignificant because it does not verify the functionality (*in* == *out*). To obtain the significant consistencies, three techniques are adopted: (1) *unit propagation delay*; (2) *unknown ignorance*; and (3) *false-enumeration ignorance*. *Unit propagation delay* means all clauses can be *true* or *false* must be inferred first before conduct unit propagation. *Unknown ignorance* means if an unknown variable is included in a *true* clause, the clause is ignored to find consistency. *False-enumeration ignorance* is the same with *unknown ignorance* but unknown variable is replaced by false variable encoding an enumeration. Rule 1 makes sure that insignificant clause like (*in*∨!*out*) can be found. Rule 2 and rule 3 will mark them as invalid clauses for consistency. The consistency can be found by searching back forward from *true* clauses and “verified” atoms (must be true for variables encoding enumerations and any values for original Boolean variables).

One important thing is that the assignments in a *consistency* are not ensured to be correct, just like the assignments in a conflict are not always wrong. A Bayesian model is proposed to analyze the relationships among the correctness of assignments, conflicts and consistencies, which is discussed in Section 3.3.1.

#### 3.2.3. MCMC-LTMS

This subsection combines the ideas of multiple-conflict and multiple-consistency together. The details of MCMC-LTMS are illustrated by pseudocodes where three routines are the most important: CHECK_CONSISTENCY, MIN_CONFLICT and MAX_CONSISTENCIES. CHECK_CONSISTENCY checks the consistency among system model *SD*, observation *obs* and candidate *ω*. MIN_CONFLICT and MAX_CONSISTENCIES find multiple minimal conflicts and multiple maximal consistencies respectively.

Algorithm 1 illuminates the framework of CHECK_CONSISTENCY for MCMC-LTMS. It applies PROPAGATE_FORWARD on *clause_set* until no propagation is made (*flag* == 0). If there exists a conflict or *false* clause in any propagation, the route returns *false*. On the contrast, it returns *true*.

**Algorithm 1.** Pseudocode of CHECK_CONSISTENCY for Multi-Conflict and Multi-Consistency-LTMS (MCMC-LTMS).CHECK_CONSISTENCY (*SD*, *obs*, *ω*)Inputs: *SD*, system description in the form of CNF *obs*, observations of observed variables *ω*, assumptions of mode variables Outputs: the consistency among *SD*, *obs* and *ω*load *obs* and *ω* *clause_set* = *SD* /* bit0 ~ unit propagate, bit1 ~ conflict or false clause, bit2 ~ true clause */ bit<3> *flag*0 = 0, *flag* = 0 ***do***  *flag* = PROPAGATE_FORWARD(*clause_set*)  *flag0* = *flag0* | *flag* ***while*** (*flag* ! = 0) *conflict* = *flag*0 & (1 << 1) ***return** conflict*? *false: true*

Algorithm 2 shows how to reason by PROPAGATE_FORWARD. For all clauses, CLAUSE_SCAN finds the *false*, *true* and *unknown* literals in clause and stores them in *false_assign*, *true_assign*, *unknown_literal*. When *true_assign* is not empty, the clause is *true*. But if the *unknown_literal* is not empty (rule 2) or there exists false assignment for variable encoding enumeration variables (rule 3), the clause is not valid for consistency. If the *unknown_literal* are more than one, the clause remains the same. If there is only one unknown literal, unit propagation is applied but delayed (rule 1). If all the literals are *false*, the clause is *false* and at least one conflict set can be obtained.

**Algorithm 2.** Pseudocode of PROPAGATE_FORWARD for MCMC-LTMS.PROPAGATE_FORWARD (*clause_set*)Inputs: clause_set, the clauses describe system Outputs: a 3-bit *flag*, bit0 ~ unit propagate, bit1 ~ conflict, bit2 ~ true clause *clause_set*, *clause_set* is replaced by the clauses remain the samebit<3> *flag* = 0 ***for** clause* in *clause_set*  CLAUSE_SCAN(*clause, false_assign, true_assign, unknown_literal*))  ***if***(*true_assign* is not empty)    *reason* = (*unknown_literal* is empty)? *true*: *false* //rule 2    PROPAGATE_TRUE_CLAUSE(*clause*, *true_assign*, *false_assign*, *reason*)//rule 3    *flag* = *flag* | (1<<2)  ***else if***(*unknown_literal.size* > 1)    remain_clause.insert(*clause*)  ***else if***(*unknown_literal.size* == 1) //rule 1    store *clause*, *false_assign*, *unknown_literal* in *fringe*  ***else***    PROPAGATE_FALSE_CLAUSE(*clause*, *false_assign*)    *flag* = *flag* | (1<<1) ***for** clause*, *false_assign*, *literal* in *fringe*  *flag* = *flag* | PROPAGATE_FRINGE_CLAUSE(*clause*, *false_assign*, *literal*)?: (1<<0): (1<<1) *clause_set* = *remain_clause* ***return** flag*

Algorithm 3 illustrates how to obtain multiple minimal conflicts when it is inconsistent. MIN_CONFLICTS searches backward from all the conflict atoms and *false* clauses, finding conflict sets recursively from down to up. If a conclusion is made by several premises, the results of the premises are combined by MIN_PRODUCT and if a conclusion is made by anyone on several premises, they are combined by MIN_PLUS. Finding the maximal consistencies are similar in Algorithm 4 except that MIN_PRODUCT and MIN_PLUS are replaced by MAX_PRODUCT and MAX_PLUS where PRODUCT means a Cartesian product of sets; PLUS means union set of sets; MIN means the minimal set; and MAX means the maximal set.

**Algorithm 3.** Pseudocode of MIN_CONFLICTS for MCMC-LTMS.MIN_CONFLICTS (*conf_atom_clause*)Inputs: *conf_atom_clause*, a conflict atom, conflict atom set, false clause or false clause set Outputs: the set of minimal conflicts*conflict* = {} ***for***
*item* in *conf_atom_clause*  ***if***(*item* is a mode atom and is *true*)    *conflict0* = {*item*}  ***else if***(*item* is a non-mode atom or clause)    *conflict0* = {}    ***for***
*i* in *item*’s support set      *conflict0* = MIN_PRODUCT(*conflict0*, MIN_CONFLICTS(*i*))  ***else***    *conflict0* = {}  *conflict* = MIN_PLUS(*conflict*, *conflict0*) ***return** conflict*

**Algorithm 4.** Pseudocode of MAX_CONSISTENCIES for MCMC-LTMS.MAX_CONSISTENCIESc(*atom*_*true_clause*)Inputs: *atom*_*true_clause*, an atom, an atom set, a true clause or a true clause set Outputs: the set of maximal consistencies*consistency* = {} ***for** item* in *atom_true_clause*  ***if***(*item* is a mode atom and is *true*)    *consistency0* = {*item*}  ***else if***(*item* is a non-mode atom or clause)    *consistency0* = {}    ***for***
*i* in *item*’s support set      *consistency0* = MAX_PRODUCT(*consistency0*, MAX_CONSISTENCIES (*i*))  *consistency* = MAX_PLUS(*consistency*, *consistency0*) ***return** consistency*

After obtaining the minimal conflicts and consistencies, the A* search algorithm is employed to generate new candidates.

### 3.3. A* Search in Diagnosis

This subsection describes how to use the A* search to generate the best candidates based on the conflicts and consistencies returned by MCMC-LTMS in two cases: single diagnosis and multiple diagnoses.

The most important idea of the A* search is to solve the diagnosis problem as an optimization problem as (15) where *g* is the evaluation function.

(15) $$\begin{array}{l} \max:\;g(\omega )\\ \mathrm{subject}\;\mathrm{to}:SD\cup obs\cup \omega |\neq ⊥ \end{array}$$

**Figure 8:**
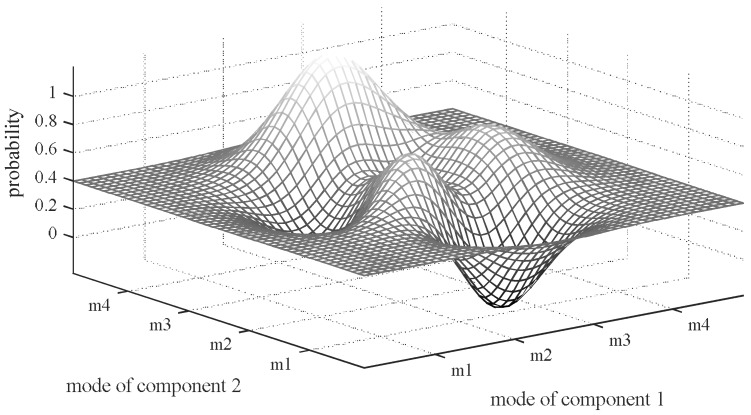
Probabilities of Different Modes Combinations.

In most cases, unfortunately, the diagnosis optimization problem for a strong-fault model is nonconvex like Figure 8 where the X-axis and Y-axis are modes of component 1 and component 2 respectively and the Z-axis is the probabilities of different mode combinations.

BFA* and CDA* solve the problem by A* search algorithm [33] based on *mutual*, *preferential independence* (MPI) [22] ensuring that the values of mode variables can be assigned one by one independently. This section discusses how to exploit priori, conflict and consistency in detail.

#### 3.3.1. Bayes in Assignments and Possible Conflicts

Conflict and consistency are different evaluation results of a redundant set. Pulido calls a redundant set a Possible Conflict (PC) [32]. This paper adopts the same terminology but uses PC to represents the unknown result of an equality check between two values (two model prediction values, or one model prediction value and one observation). Traditional methods simply assume that if there is a conflict, the candidate is invalid (false alarm probability is zero); if there is no conflict, the candidate is a diagnosis (missing probability is zero). For the discrete model, the first rule is right according to the definition of consistency-based diagnosis [29]. However, the second rule is not absolutely right for a strong-fault model. One of the most famous examples is “Stuck Fault” [20] which means a component gets stuck on a mode in spite of commands. When the component is in the mode *m*, there will be no conflict even the candidate set the component in “stuck *m*.” Although the candidate can be lost because it contains more fault number compared with the candidate assuming the component is in the mode *m*, the oversimplified probability model cannot help us to evaluate the cost if we want to set some mode variables different values from a consistency set. Because if missing alarm is zero, the assignments in a consistency must be right. However, the statement can be wrong. This part analyzes the influence relations between mode variable assignments and possible conflicts using the Bayesian method and proposes a new simplified probability model, assuming that: (1) each mode assignment influences each involved PC independently; (2) false alarm probability is zero (due to the definition of consistency-based diagnosis); and (3) missing alarm probability is a small constant.

**Figure 9:**
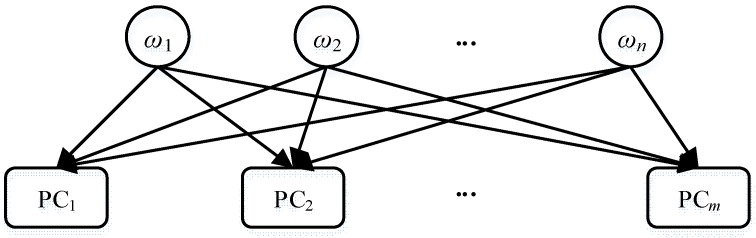
Structure of Mode Variables and Possible Conflicts.

The bipartite graph in Figure 9 illustrates the influence relationships between mode variables and PCs, where *ω_i_* is the assignment for a mode variable and PC*_i_* is a possible conflict. Possible conflict PC = 1 means the PC is consistent (consistency) and PC = 0 means inconsistent (conflict). *ω_i_* = 1 means the assignment is *correct* and *ω_i_* = 0 means the assignment is *wrong*. Without confusion, *ω_i_* is also used to represent the assigned mode value of component *i*. In the worst case, any assignment is involved in all possible conflicts as in Figure 9. However, usually one possible conflict only involves several assignments. The *k_j_* components involved in the *j*th possible conflict PC*_j_* are denoted by $\omega_{j1},\omega_{j2}\cdots \omega_{jk_{j}}$. Based on the structure in Figure 9 and results of PCs, the probability of mode assignments is evaluated by (16) to find the best candidate.

(16) $$\begin{array}{ll} P(\omega_{1},\omega_{2}\cdots \omega_{n}|{\mathrm{PC}}_{1},{\mathrm{PC}}_{2}\cdots {\mathrm{PC}}_{m}) & =\frac{P(\omega_{1},\omega_{2}\cdots \omega_{n},{\mathrm{PC}}_{1},{\mathrm{PC}}_{2}\cdots {\mathrm{PC}}_{m})}{P({\mathrm{PC}}_{1},{\mathrm{PC}}_{2}\cdots {\mathrm{PC}}_{m})}\\ & =\prod_{i=1}^{n}P(\omega_{i})\\ & \;\times \prod_{j=1}^{m}P({\mathrm{PC}}_{j}|\omega_{j1},\omega_{j2}\cdots \omega_{jk_{j}})\\ & \;\times \frac{1}{P({\mathrm{PC}}_{1},{\mathrm{PC}}_{2}\cdots {\mathrm{PC}}_{m})} \end{array}$$

where $\prod_{i=1}^{n}P(\omega_{i})$ is the priori probability of assignments, $\prod_{j=1}^{m}P({\mathrm{PC}}_{j}|\omega_{j1},\omega_{j2}\cdots \omega_{jk_{j}})$ is the conditional probability mentioned in (7) and $P({\mathrm{PC}}_{1},{\mathrm{PC}}_{2}\cdots {\mathrm{PC}}_{m})$ is the normalization factor. Finding the best candidate means to solve optimization problem (17) where $\vec{\omega }={\left[\omega_{1},\omega_{2}\cdots \omega_{n}\right]}^{T}$.

(17) $$\max:g(\vec{\omega })=\prod_{i=1}^{n}P(\omega_{i})\;\times \prod_{j=1}^{m}P({\mathrm{PC}}_{j}|\omega_{j1},\omega_{j2}\cdots \omega_{jk_{j}})$$

It is difficult to solve (17) because the probabilities in this formula are coupled with the real structure of system. To provide a general diagnosis method, this paper assumes that each mode assignment *ω* effects the probability independently and the simplified distribution model for $P({\mathrm{PC}}_{j}|\omega_{j1},\omega_{j2}\cdots \omega_{jk_{j}})$ is shown in Table 1 where *ε* (missing alarm probability) is positive number close to zero. If a fault may not cause a conflict, *ε* should be larger.

The distribution in Table 1 indicates that: when all the assignments in a PC is *correct* (*f* = 0), PC must be consistent (*P*(PC = 1) = *ε*^0^ = 1 and *P*(PC = 0) = 1 − *ε*^0^ = 0); when one assignment is wrong, PC is probability to be a conflict (*P*(PC = 1) = *ε* and *P*(PC = 0) = 1 − *ε*); when there are multiple assignments are wrong, *ε^f^* and 1 − *ε^f^* are the probability of consistency and conflict respectively. Briefly, if PC*_j_* = 0, there must be at least one wrong assignment in $\omega_{j1},\omega_{j2}\cdots \omega_{jk_{j}}$; if PC*_j_* = 1, the probability of any *ω* = 1 in $\omega_{j1},\omega_{j2}\cdots \omega_{jk_{j}}$ is much higher.

To avoid numerical problem, (17) is converted into a cost-based minimization optimization problem (18) where *f_j_* is the number of different assignments between new candidate and consistency or conflict (assuming new candidate is *correct*, the different assignments in consistency or conflict are wrong assignments), *n* is the number of components and *m* is the number of PCs.

(18) $$\begin{array}{ll} \min:c(\vec{\omega }) & =-\ln(\prod_{i=1}^{n}P(\omega_{i})\;\times \prod_{j=1}^{m}P(PC_{j}|\omega_{j1},\omega_{j2}\cdots \omega_{jk_{j}}))\\ & =-\sum_{i=1}^{n}\ln P(\omega_{i})-\sum_{j=1}^{m}\ln P(PC_{j}|\omega_{j1},\omega_{j2}\cdots \omega_{jk_{j}})\\ & =\underset{priori\;cost}{\underset{︸}{\sum_{i=1}^{n}\left(-\ln P(\omega_{i})\right)}}+\underset{consistency-break\;cost}{\underset{︸}{(-\ln\varepsilon )\sum_{j=1,\;PC_{j}=1}^{m}f_{j}}}+\underset{conflict-solve\;cost}{\underset{︸}{\sum_{j=1,\;PC_{j}=0}^{m}\left(-\ln(1-\varepsilon^{f_{j}})\right)}} \end{array}$$

The cost is composed of three parts: priori cost which is determined by the priori probability, consistency-break cost which is the cost to assign mode values different from the assignments in consistencies and conflict-solve cost which is the similar to break consistency cost but assigns different values from conflicts. Equation (18) indicates that: (1) any different assignment from consistency results in a high punishment in the cost; (2) If there is no different assignment from any conflict, the cost will be positive infinite and the more different assignments from the conflicts, the less the cost is. To minimize the cost function, the consistency-break cost prefers to reserve the assignments in all consistencies and the conflict-solve cost prefers to change all assignments in conflicts. With the same priori probability, different assignments from consistency will increase cost but different assignments from conflict will reduce cost. However, in most cases, the more different assignments from conflict, the priori cost will be larger. Best candidate should make a balance among priori, breaking consistency and solving conflict.

**Figure 10:**
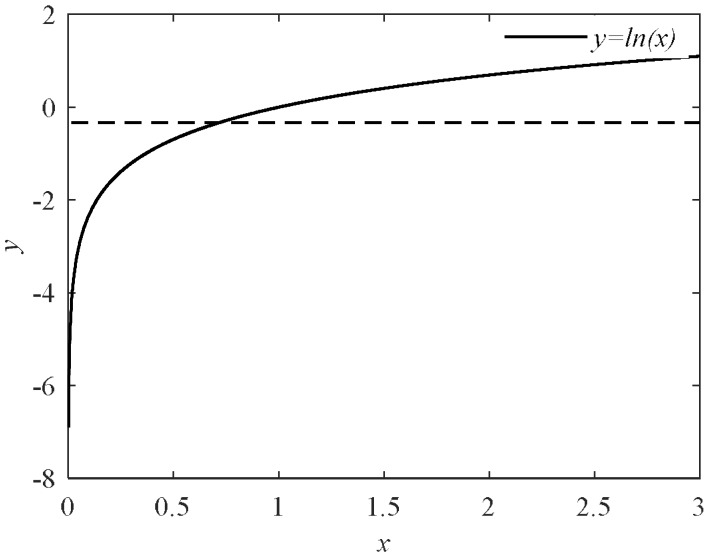
*y* = ln(*x*).

The three parts of cost function in (18) contribute differently to the whole cost. Figure 10 shows the curve of natural logarithm function *y* = ln(*x*) in which *y* is close to zero when *x* is close to 1 and *y* is close to negative infinite when *x* is close to zero. For priori cost, usually *P*(*ω_i_*) for normal mode is close to 1 but close to 0 for fault mode. When finding a candidate containing fault modes, priori cost is not a small quantity. In consistency-break cost, because *ε* is a positive number close to zero, ln*ε* is not a small quantity and when assignments in candidate are different from those in consistency, consistency-break cost contributes a lot. And consistency-break cost is zero if the assignments in consistencies keep the same. For conflict-solve cost, when assignments in conflicts are not changed, it is infinite, however it is a small quantity when some assignments in conflicts are changed and conflict- solve cost does not decrease much when *f* increases.

So, (18) can be approximated by (19).

(19) $$cost(\vec{\omega })=\{\begin{array}{c} \infty ,\;\mathrm{there}\;\mathrm{exists}\;\mathrm{unsolved}\;\mathrm{conflict}\\ \underset{priori\;cost}{\underset{︸}{\sum_{i=1}^{n}\left(-\ln P(\omega_{i})\right)}}+\underset{consistency-break\;cost}{\underset{︸}{(-\ln\varepsilon )\sum_{j=1,\;PC_{j}=1}^{m}f_{j}}},\;\mathrm{else} \end{array}$$

The only problem for (19) is that, to construct a heuristic function for A* search, consistency-break cost should be in the form of a sum according to component, rather than a sum according to PC. Fortunately, it is very easy to convert. The converted formula is shown in (21).

#### 3.3.2. Single Diagnosis—CCDLSA*

With the MPI property, finding a single diagnosis is relatively simple. The first best candidate *ω* is generated by assigning each mode variable the most likely value (for example, all components are normal) according to the prior probability. If there is no conflict found, *ω* is a diagnosis and else new candidate must be generated based on the information returned by MCMC-LTMS.

Williams B. C. et al. [22] employs kernel constitutes to find the next best candidate. The core idea behind kernel constitutes is that even if a component is set as fault by mistake, it will not cause more conflicts. However, the diagnosis in solution space for a strong-fault model is nonmonotonic which may cause the expansion of conflict. For example, assuming component B is faulty in Figure 7. If detect a conflict at *out*1 (conflict set {A, B}) and the kernel constitute algorithm takes {A} as the candidate by mistake. For a weak-fault model, the only mode of A can be *fault* and the conflict set will still be {A, B} because setting component A as fault predicts no wrong value at the output of A. However, for a strong-fault model, a specific fault mode must be assigned to component A and a fault mode will cause a wrong output for component A and there will be two conflict sets {A, B} and {A, C}. The expansion of conflict causes “combinatorial explosion” and a strong-fault model cannot adopt kernel constitutes method.

In this subsection, we propose a Conflict and Consistency Directed Local Sequential A* search (CCDLSA*) to solve this problem where “local” means only components involved in conflicts are searched and “sequential” means new possible fault components are added sequentially. A* algorithm is employed in the search.

(20) $$<P(\omega_{i}),f_{i}>$$

Each node in the search tree is labelled by (20) where *P*(*ω_i_*) is the priori probability, *f_i_* is the occurrence number of component *i* in all consistencies in which *ω_i_* is different from the assumption. The cost of the assignments is defined by (21) if all the conflicts are solved.

(21) $$\sum_{i=1}^{n}\left(-\ln P(\omega_{i})+(-\ln\varepsilon )f_{i}\right)$$

where *n* is the number of variables in the assignments. If the assignment does not solve all the conflicts, its cost is positive infinite.

**Figure 11:**
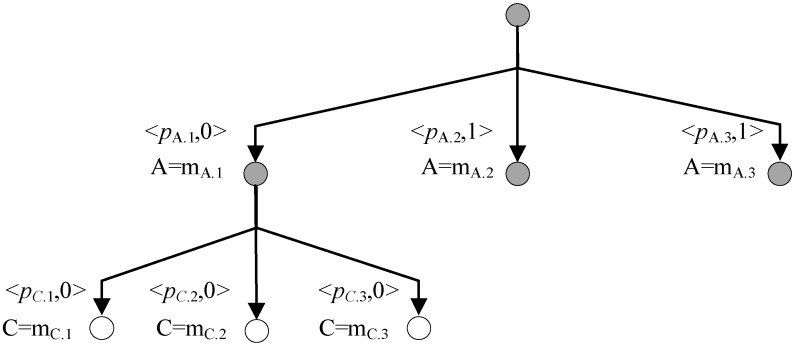
Search Tree of Conflict and Consistency Directed Local Sequential A* search (CCDLSA*)*.

Take the system in Figure 7 as an example. Assume each component has three modes. {A = m_A.1_, B = m_A.1_} is a consistency and {A = m_A.1_, C = m_C.1_} is a conflict. The search tree is illustrated in Figure 11 where the algorithm prefers to believe that A’s mode is m_A.1_ because it does not break any consistency. Algorithms 5 and 6 illustrate the proposed method.

Algorithm 5 shows the framework of CCDLSA*. First, all the components are assumed to be normal. If it is not consistent with *SD* and *obs*, the minimal conflict and maximal consistency sets are obtained. FAULT_COMPONENT finds possible fault components which are assigned as *normal* in a conflict set and new possible fault components are pushed back to vector *fault_components*. A*_BEST_CANDIDATE finds the next best candidate whose consistency is further checked by CHECK_CONSISTENCY. The procedure loops till a consistent diagnosis *ω* is found. Because the addition of consistencies will change the cost of existing nodes, only consistencies in the first consistency check are utilized.

**Algorithm 5.** Pseudocode of CCDLSA*.CCDLSA*(*SD*, *obs*)Inputs: *SD*, system description in the form of CNF *obs*, observations of observed variables Output: *ω*, the diagnosis consistent with *SD* and *obs**ω* = all components are normal ***if***(!CHECK_CONSISTENCY(*SD*, *obs*, *ω*))  *atom*_*true_clause* = consistency variables or valid true clauses  *conf_atom_clause* = conflict variables or valid false clauses  *conflicts* = {}, *fault_components* = empty vector  *consistencies* = MAX_CONSISTENCIES(*atom_true_clause*)  ***do***    *conflicts* = *conflicts* + MIN_CONFLICTS (*conf_atom_clause*)    push back FAULT_COMPONENT(*conflicts*) - *fault_components* into *fault_components*    *ω* = A*_BEST_CANDIDATE(*conflicts*, *consistencies*, *fault_components*)  ***while***(!CHECK_CONSISTENCY(*SD*, *obs*, *ω*)) ***return** ω*

Algorithm 6 shows the kernel algorithm A*_BEST_CANDIDATE. A *queue* is first initialized by empty set {}. Then the node *ω* with minimal cost in *queue* is always popped out. If *ω* does not assign values for all components in *fault_components*, node *ω* is expanded by EXPAND where cost is defined by (21). If it does, *ω* is the best candidate after adding other default assignments by ADD_DEFAULT.

CCDLSA* is efficient because both conflict and consistency are employed to generate best candidate and the search space is restricted by the possible fault components. However, it is not good at obtaining multiple diagnoses because maybe there exist no enough diagnoses in local space.

**Algorithm 6.** Pseudocode of A*_BEST_CANDIDATE for CCDLSA*.A*_BEST_CANDIDATE (*conflicts*, *consistencies*, *fault_component*)Inputs: *conflicts*, the set of all conflicts *consistencies*, the set of all consistencies   *fault_components*, the set of possible fault components Output: *ω*, the best candidateadd {} into *queue* **do**  *ω* = pop *queue*  ***if***
*ω* does not assign values for all components in *fault_components*    EXPAND(*ω*, *conflicts*, *consistencies*)  ***else***    *break* ***while***(1) *ω* = ADD_DEFAULT(*ω*) ***return** ω*

#### 3.3.3. Multiple Diagnoses—CCDGA*

The previous subsection proposes a novel focused search diagnosis method to get one diagnosis and this subsection discusses how to get multiple ones. As shown in Figure 8, the search space is nonconvex. It is likely to fall into a local space and fail in getting enough diagnoses. This subsection discusses how to solve this problem by Conflict and Consistency Directed Global A* search (CCDGA*).

**Figure 12:**
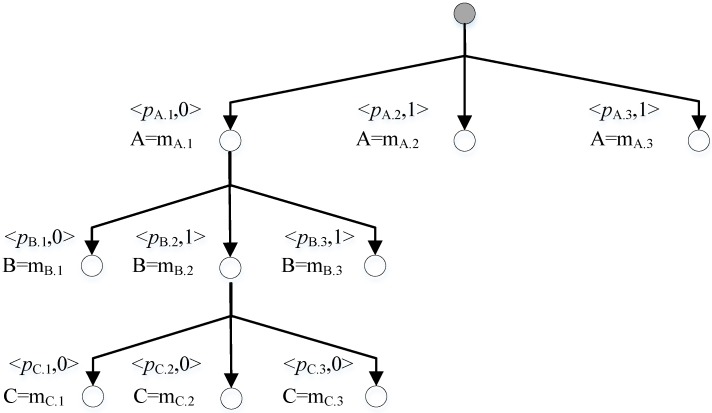
Search Tree of Global based A* Search.

Briefly, CCDGA* expands the search space from possible fault components to all the components. Figure 12 illustrates the search tree of a global based diagnosis, for example used in 0. The basic algorithm shown in Algorithm 7 is similar to the one in Algorithm 5 except that the initial assignments are obtained from the search tree and the loop stops until enough diagnoses are obtained. A*_BEST_CANDIDATE in Algorithm 7 is the same with Algorithm 6 except that *fault_components* are replaced by all components. CCDGA* can also be used to obtain single diagnosis but CCDLSA* outperforms it because of the less search space.

**Algorithm 7.** Pseudocode of CCDGA*.CCDGA*(*SD*, *obs*, *k*)Inputs: *SD*, system description in the form of CNF *obs*, observations of observed variables   *k*, the number of diagnosis want to obtain Output: *Ω*, the diagnosis set consistent with *SD* and *obs**conflicts* = {}, *consistencies* = {} ***do***  *ω* = A*_BEST_CANDIDATE(*conflicts*, *consistencies*, *all_components*)  *atom*_*true_clause* = consistency variables or valid true clauses in the first consistency check  *conf_atom_clause* = conflict variables or valid false clauses  ***if***(!CHECK_CONSISTENCY(*SD*, *obs*, *ω*))    *conflicts t* = *conflicts* + MIN_CONFLICTS (*conf_atom_clause*)    *consistencies* = MAX_CONSISTENCIES(*atom*_*true_clause*) in the first consistency check  else    add *ω into Ω* ***while***(size of *Ω < k*) ***return** Ω*

## 4. Algorithm Analysis

This section analyzes methods proposed in Section 3.

### 4.1. MCMC-LTMS

This subsection discusses completeness, coverage and complexity of MCMC-LTMS.

• Completeness

Completeness is defined as the ability to predict all the outputs based on enough inputs. As MCMC-LTMS employs unit propagation as the reason method, it is incomplete in theory. However, “A strong-fault model” makes *SD* a special subset of propositions. Let’s first look at why unit propagation is incomplete. Assuming *A* and *B* are two Boolean variables.

(22) (A∨B)∧(!A∨B)

(23) (A∨!A)∧B

Then (22) and (23) are the same proposition in different forms. (22) is a CNF and apparently unit propagation cannot be applied. But (23) tells us that *B* must be *true* because (*A*∨!*A*) must be true. As CNF is the standard form of unit propagation, unit propagation is incomplete. But the problem no longer exists for a strong-fault model.

To be brief, assume that the behavior of each mode is described by Boolean variables in the form of (24)

(24) $$m\Rightarrow (P_{IN}\Rightarrow l_{out})$$

where *m* is the mode variable, *P_IN_* is a proposition composed of input variables and *l_out_* is the literal of output variable *out*.

(25) $$\left({C^{1}}_{m,IN}\vee {l^{1}}_{out})\wedge ({C^{2}}_{m,IN}\vee {l^{2}}_{out})\wedge \cdots \wedge ({C^{n}}_{m,IN}\vee {l^{n}}_{out}\right)$$

Equation (25) is the CNF form of (24) where *C^i^_m,IN_* is a clause composed *m* and input variables and *l^i^_out_* is a literal of *out*. *m* will be assumed in diagnosis. Inputs are observed or the outputs of other components. If the all the inputs of this component are observed, (*C^i^_m,IN_*∨*l^i^_out_*) is a *true*/*false* clause or the value of *out* can be inferred because the value of *C^i^_m,IN_* is determined. If some inputs of this component are not observed, they are sure to be inferred from other components. So, the value of all unknown non-mode variables will be inferred by unit propagation.

As a conclusion, MCMC-LTMS is complete for a strong-fault model.

• Coverage

The coverage discusses if MCMC-LTMS is able to find conflicts covering all fault components. Before analysis, an assumption is made: fault components will surely cause at least one conflict. The assumption means a fault mode will always predict a different output from the normal mode and the outputs of multiple faults will never be counteracted. Although sometimes the assumption is not satisfied, it is necessary to analyze the coverage of MCMC-LTMS theoretically.

When a system is abnormal, there are two methods to find conflict: difference between predicted value and observed value of output and *false* clause. For the first case, all the outputs are predicted and all the functionalities of the system are verified, which ensures MCMC-LTMS cover all possible fault components, For the second case, the false clause will not stop the propagation of false predicted values. More components may be involved but the real fault component will never be missed.

Under the assumption, MCMC-LTMS can cover all possible fault components. If the assumption is not satisfied, the coverage is not ensured.

• Complexity

The operation of MCMC-LTMS is composed of two steps: (1) reasoning to get the values of unknown variables and (2) backtracking to find all the conflicts and consistencies.

In the first step, because our object systems are described by a strong-fault model, all the unknown variables can be inferenced by unit propagation and the complexity is *O*(*na*) where *n* is the number of clauses in a CNF model and *a* is the time to execute one unit-propagation.

In the second step, if *m* conflicts and consistencies are found in the whole and *b* is the time to find one conflict or consistency. The time complexity is *O*(*mb*).

As a conclusion, the complexity is *O*(*na*+*mb*). As a contrast, the complexity of standard LTMS is O(*na*+*mb*) where m is 0 or 1 because it only finds the support set of one conflict. Although MCMC-LTMS seems less efficient, it has the same complexity when there is no conflict and MCMC-LTMS saves many reasoning steps when there exist conflicts because it merges information from multiple conflicts.

### 4.2. A* Search in Diagnosis

#### 4.2.1. Single Diagnosis—CCDLSA*

• Correctness

We define the correctness of proposed algorithm as the ability to detect conflict if there exists one and the ability to find a consistent diagnosis. Sometimes a fault will not cause conflict but the problem can only be solved by improving model rather than algorithm.

For a strong-fault model, MCMC-LTMS is complete. It is able to find at least one conflict if exist. Although MCMC-LTMS cannot cover all possible fault components in the first consistency check when the assumption is not satisfied, the any possible fault components missed in the first check will be added to the search space in the next loops. CCDLSA* enumerates all the possible combinations and the real assignments must be in them. So CCDLSA* is sure to terminate and get the diagnosis.

• Complexity

The space and time complexity of CCDLSA* is the same and is related to the number of possible combinations. In the worst case, CCDLSA* must enumerate all the possible combinations which indicates that the complexity is *O*(*m^n^*) where *m* is the average assignment number and *n* is the fault component number. And if consistency eliminates normal components from fault components correctly, the complexity is reduced.

• Optimality

The A* search is optimal when the estimated cost of unassigned mode variables in heuristic function is not overestimated. The heuristic function used in this paper set the estimated cost as zero which can never be over the real cost. Therefore, CCDLSA* is optimal.

• Robustness

To be optimal, the A* search usually conducts multiple backtracking when it finds that the current node is not optimal. If the priori distribution of mode variable is sharp, which means that the probabilities of different values for one mode variable vary a lot, it is easy to distinct different combinations by priori probabilities and less backtracking occurs. If the prior distribution is flat, the priori probabilities of different are similar to each other and more backtracking occurs.

In a summary, if the priori distributions of mode variables are sharp, the CCDLSA* is robust. However, if the priori distributions are flat, the robustness is not ensured.

#### 4.2.2. Multiple Diagnoses—CCDGA*

• Correctness

The correctness of CCDGA* is similar to CCDLSA*. Because it traverses the solution space for all the components, it is sure to find all the possible diagnoses.

But if our expected diagnoses number exceeds the existing ones, CCDGA* will traverse the whole solution space and usually not terminate in acceptable time.

• Complexity

The complexity of CCDGA* is also similar to CCDLSA* except that *n* is the number of all components.

• Optimality

Due to the same analysis for CCDLSA*, CCDGA* is optimal.

• Robustness

When there exit enough diagnoses, robustness of CCDGA* is the same with CCDLSA*: if the priori distributions of mode variables are sharp, the CCDGA* is robust. However, if the priori distributions are flat, the robustness is not ensured. However, if there are fewer diagnoses than the expected number, CCDGA* will traverse the whole search space, which is too costly.

## 5. Case Study

### 5.1. Model Introduction

**Figure 13:**
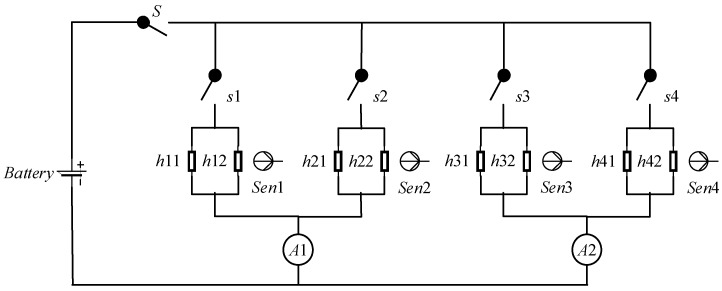
Heat Control System.

Figure 13 shows a heat control system composed of battery, switch, heater, sensor and ammeter in satellite. The battery offers power so that the heater can keep other equipment warm and operating well. There is a main switch *S* to control all the heaters and four switches *s_i_* (*i* = 1, 2, 3, 4) to control four heaters. Heater *h_i_* (*i* = 1, 2, 3, 4) is composed of two resistors *h_i_*_1_ and *h_i_*_2_. Four sensors *Sen_i_* (*i* = 1, 2, 3, 4) are used to monitor the heating efficiency. Two ammeters *A*1 and *A*2 monitor the current at two points.

**Figure 14:**
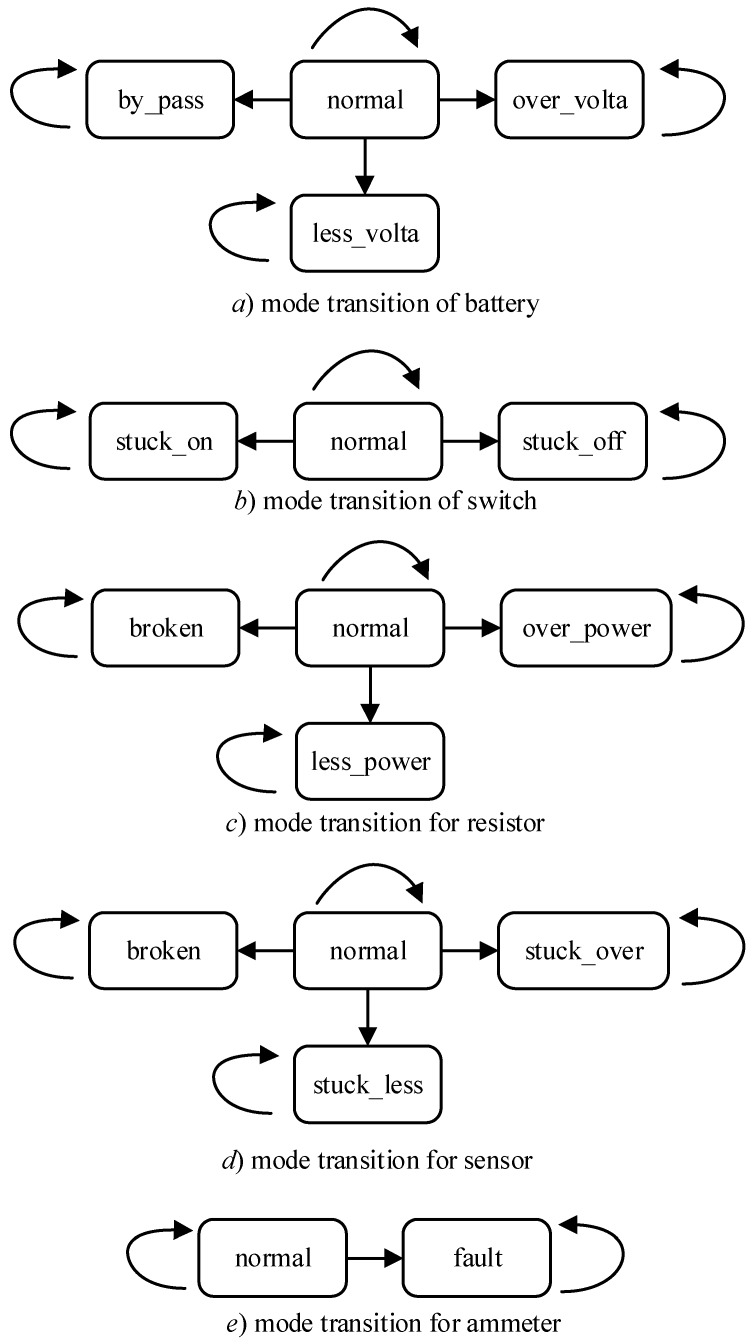
Mode Transitions.

The modes of all the components are shown in Figure 14 in which normal modes can transfer to both normal modes and fault modes but fault modes can never transfer to normal modes. Table 2 illustrates the behaviors of each component in different modes in details.

The system in Figure 14 is composed of 20 components where there are 4 sensors, 5 switches, 8 resistors, 2 ammeters and 1 battery in whole. The search space is 4 × 3^5^ × 4^8^ × 4^4^ × 2^2^ = 65,229,815,808 ≈ 6.5 × 10^10^ which is too huge to search by traversing. After encoding, the system is described by Boolean variables and there are 1059 constraint clauses (model) over the system which are also a huge challenge to MCMC-LTMS. The system is employed to demonstrate the proposed methods in the next two subsections where all the experiments are conducted on a i7-8550u, 8G RAM laptop.

### 5.2. MCMC-LTMS

This subsection demonstrates the ability of MCMC-LTMS to find conflict and consistency. The 11 observations are shown in Table 3 where cS is the command for switch S, cs1~cs4 are the commands for s1~s4, t1~t4 are the output values of Sen1~Sen4 and c1 and c2 are the monitored currents of A1 and A2.

Table 4 shows the conflicts and consistencies found by MCMC-LTMS where there are 1 conflict and 5 consistencies. Based on the structure in Figure 13, it is obvious that the conflict locates the fault component in sensor 3 and its upstream components. The first three consistencies mean that branches 1, 2 and 4 including three sensors are normal. The last two consistencies extend the normal scope to h31, h32, A1 and A2. The most interesting consistency is {battery, S, s3, s4, h31, h32, h41, h42, A2} in which h31 and h32 may be in fault mode *less_power* in theory but because the predicted outputs at A1 and A2 are consistent with observed values, h31 and h32 are included in consistency.

The result in Table 4 indicates that MCMC-LTMS can effectively analyze system behaviors and find conflict and consistency set based on the structure information hidden in encoded model.

### 5.3. A* Search in Diagnosis

#### 5.3.1. Single Diagnosis

Best First A* search (BFA*), Conflict Directed A* search (CDA*, multiple fault version, single fault version cost too much time), CCDLSA* (*ε* = 1.0e-2) and CCDGA* (*ε* = 1.0e-2) were evaluated to find one diagnosis for the same scenario in Table 3.

**Figure 15:**
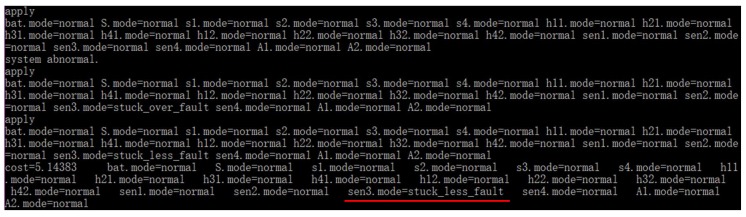
Diagnosis Process of CCDLSA* for Single Diagnosis.

Figure 15 provides the diagnosis process of CCDLSA* where the algorithm tried 3 candidates in all and the diagnosis results showed that sensor *Sen*3 was in *stuck_less* fault mode and other components were normal. The correctness of the diagnosis can be easily verified according to system structure and component behavior. To be brief, the diagnosis processes of other algorithms are not given in this paper. All the left 3 algorithms had the same diagnosis results but different diagnosis time and tried candidates number.

To compare the performances of different methods, BFA* is adopted as the criterion. The “Ratio,” defined by (26), in the next 4 tables are the percentages of “algorithm” based on BFA* for different “*value*”s.

(26) $$Ratio(\mathrm{algorithm})=\frac{value\;of\;\mathrm{algorithm}}{value\;of\;BFA*}\times 100\%$$

**Figure 16:**
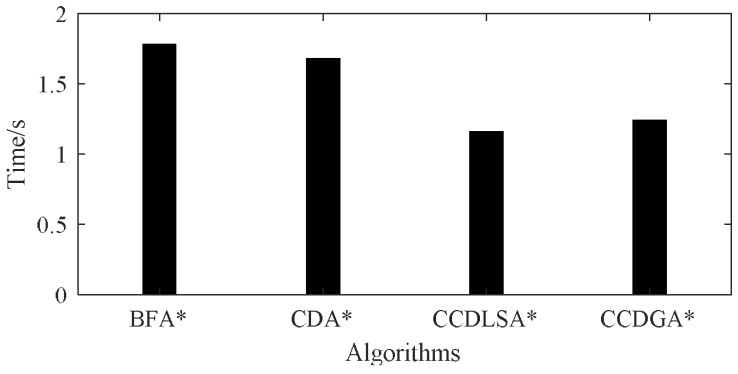
Average Diagnosis Time for BFA*, CDA*, CCDLSA* and CCDGA*.

The time for 10 experiments is shown in Table 5 where “Ave” means the average time and “Ratio” means the percentages of diagnosis time based on BFA*. We can see that the time for 10 experiments fluctuates slightly around the average time which is also shown in Figure 16. The small fluctuation implies that all of the 4 algorithms are stable and proves the robustness of the proposed methods. In average, CCDLSA* and CCDGA* cost less time than BFA* and CDA*. Due to the smaller search space of CCDLSA*, it was slightly better than CCDGA*.

The numbers of nodes for search are shown in Table 6 where “Tried Candidates” means the number of candidates tried by the algorithm, “Expanded Nodes” means the number of nodes expanded in the search tree and “Node in Queue” means the number of the leaf nodes in the search tree when the diagnosis is finished. “Num” means the number of nodes and the percentages “Ratio” beside the numbers are the ratios of their node numbers and the node number for BFA*. Although CDA* tried fewer candidates than BFA*, the numbers of nodes expanded and in queue were still much larger than CCDLSA* and CCDGA*. CCDLSA* tried the least candidates, expanded least nodes and left least nodes in the queue as well.

Based on the analysis about Table 5 and Table 6 and Figure 16, we can see that CCDLSA* and CCDGA* can avoid invalid node expansion effectively. This feature helps the two proposed algorithms consume less memory space and time to diagnose.

#### 5.3.2. Multiple Diagnoses

This experiment evaluates BFA*, CDA* and CCDGA* (*ε* = 1.0e-2) in finding 3 diagnoses.

**Figure 17:**
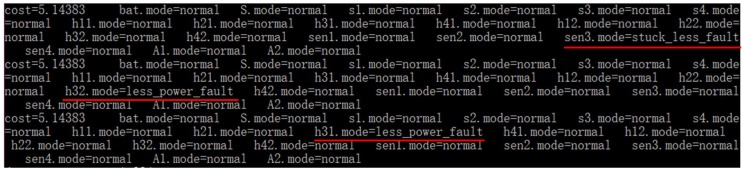
Diagnosis Results of CCDGA* for 3 diagnoses.

The diagnosis results of CCDGA* are shown in Figure 17 where the three diagnoses were: (1) sensor *Sen*3 was in *stuck_less* fault mode; (2) heater *h*32 was in *less_power* fault mode and (3) heater *h*31 was in *less_power* fault mode (all other components in the three diagnoses were normal). All the three diagnoses could explain the observation. BFA* and CDA* gave the same results, which were not shown in this paper to avoid redundancy.

**Figure 18:**
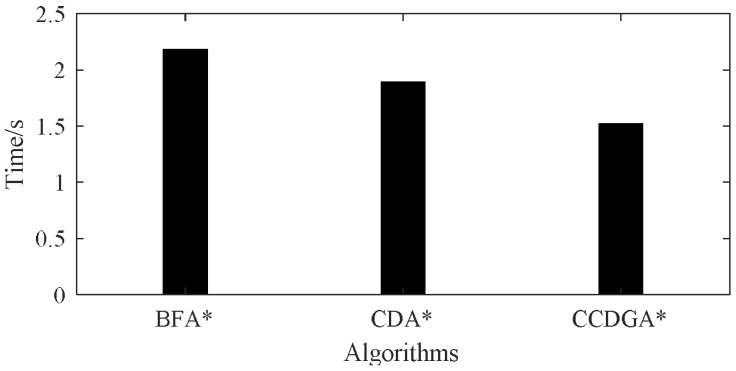
Average Diagnosis Time for BFA*, CDA* and CCDGA*.

The diagnosis times for 10 experiments are given in Table 7, the average time is illustrated in Figure 18 and Table 8 shows the statistic information of nodes for search.

The statistical data in Table 7 and Table 8 and Figure 18 are similar to the data for single-diagnosis. CCDGA* was robust in the test, tried the least candidates, expanded the least nodes and kept the least node in the queue. Although CDA* also just tried a small number of candidates, the nodes expanded and reserved in the queue approaches the numbers of BFA*. In general, CCDGA* has both space and time advantages over CDA* and BFA*.

The section evaluates each module in the proposed methods comprehensively. Section 5.2 shows that MCMC-LTMS can not only check the consistency between system model, candidate and observation but also effectively find conflict and consistency set even though the novel LTMS does not explicitly utilize the system structure model. The two algorithms based on MCMC-LTMS are accessed in Section 5.3 where both single diagnosis and multiple diagnoses manifest that the proposed search methods can accurately skip improbable candidates based on the results from MCMC-LTMS. Compared with BFA* and CDA*, both CCDLSA* and CCDGA* can significantly reduce the space and time cost in diagnosis.

## 6. Conclusions and Future Work

This paper presents research on reasoning and search in MBD for a strong-fault model. The greatest contribution is the proposition of *consistency* in MBD. In fact, the PC corresponds to the residual in Fault Detection and Identification (FDI) [11]. Evaluating a residual as 1 amounts to a PC is verified as a conflict. In the opposite, the PC is proven to be a consistency when the residual is 0. The proposition of *consistency* makes MBD and FDI more consistent.

To obtain consistency when reasoning over discrete model, this paper proposed a novel MCMC-LTMS which is able to obtain multiple conflicts and consistencies in one reasoning process. In theory, MCMC-LTMS is complete for a strong-fault model and can cover all fault components in most cases. In the experiment, MCMC-LTMS found the only minimal conflict and all the maximal consistencies without using system structure information explicitly.

Faults can be isolated efficiently if the information in conflict and consistency is fully utilized. By Bayesian analysis and the introduction of missing alarm probability, a more exquisite probability model was proposed. Based on the probability model, *P*(*obs*|*mode*) can be evaluated more accurately.

An approximate version of *P*(*obs*|*mode*) provided by the novel probability model are used to construct the heuristic function for A* search. With the proposed heuristic function, two A* search algorithms are designed for single diagnosis and multiple diagnoses respectively. It has been proven theoretically that although the two algorithms may traverse the whole search space in the worst case, the diagnoses are correct and optimal for a strong-fault model. For the sharp priori distribution, both algorithms are robust except that when expecting too many diagnoses, CCDGA* is too time costly. The search algorithms may perform badly for the flat priori distribution. The experiment showed that all the tested algorithms were robust and could give the correct diagnoses but both CCDLSA* and CCDGA* expanded fewer nodes in the search, tried fewer insignificant candidates and cost less time. Because CCDLSA* has a smaller search space than CCDGA*, CCDLSA* performed better in single diagnosis. Specifically, using BFA* as the criterion, for single diagnosis, CCDLSA* and CCDGA* reduced time by 33.33% and 28.74% respectively. However, CDA* just reduced by 3.45%; for 3 diagnoses, CCDGA* reduced time by 30.28% and CDA* only reduced by 13.3% as a comparison. Besides, CCDLSA* and CCDGA* also significantly outperformed CDA* in tried candidates, expanded nodes and nodes in queue, which indicated that the proposed algorithms cost less memory space.

Theoretical analysis and experiment manifest that the proposed diagnosis methods for a strong-fault model are effective and efficient. For a sharp priori distribution, both CCDLSA* and CCDGA* are suitable for single diagnosis but CCDLSA* is better. For multiple diagnoses, CCDGA* is effective with sharp priori distribution and a proper expected diagnosis number.

Currently, the search algorithms can only employ the consistencies found in the first iteration because the weights of nodes may change in the search tree based on new consistencies which causes reconstruction of the search tree. How to make use of consistencies incrementally will be studied in the future.

## Figures and Tables

**Table 1:** Simplified Distribution of Mode Variable Assignments and Possible Conflicts.

Mode Assignments	PC*_j_*	Conditional Probability
***f*** **is the number of *wrong* assignments**	1	*ε^f^*
	0	1 − *ε^f^*

**Table 2:** Components, Modes and Behaviors.

Component	Mode	Behavior
**battery**	*normal*	The output voltage is normal.
	*by_pass*	The output voltage is zero.
	*less_volta*	The output voltage is lower than normal.
	*over_volta*	The output voltage is higher than normal.
**switch**	*normal*	When the switch is on, output is equal to the input. Zero when off.
	*stuck_on*	The switch keeps on ignoring the command.
	*stuck_off*	The switch keeps off ignoring the command.
**resistor**	*normal*	If the input voltage is zero, there is no heat and output current is zero. If the input voltage is less, power is less and output current exists. If the input voltage is normal, power is normal and output current exists. If the input voltage is over, power is over and output current exists.
	*less_power*	If the input voltage is zero, there is no heat and output current is zero. Otherwise, power is less and output current exists.
	*over_power*	If the input voltage is zero, no heat and output current is zero. Otherwise, power is less and output current exists.
	*broken*	There is no heat and output current is zero.
**sensor**	*normal*	If both resistors generate no heat, output temperature is none. If both resistors generate heat normally or one is less power but the other is over power, output temperature is normal. If one resistor generates heat over power and the other one is normal or over power, output temperature is over. In other cases, output temperature is less.
	*stuck_less*	Output temperature is less.
	*stuck_over*	Output temperature is over.
	*broken*	Output temperature is broken.
**ammeter**	*normal*	The output is the number of input currents.
	*fault*	The output is always zero.

**Table 3:** Observations for Heat Control System.

Variable	cS	cs1	cs2	cs3	cs4	t1	t2	t3	t4	c1	c2
Value	true	true	true	true	true	norm	norm	less	norm	four	four

**Table 4:** Conflict and Consistency Found by MCMC-LTMS.

Type	Set
Conflict	{battery, S, s3, h31, h32, sen3}
Consistency	{battery, S, s1, h11, h12, sen1}
	{battery, S, s2, h21, h22, sen2}
	{battery, S, s4, h41, h42, sen4}
	{battery, S, s1, s2, h11, h12, h21, h22, A1}
	{battery, S, s3, s4, h31, h32, h41, h42, A2}

**Table 5:** Single Diagnosis Time(s) for BFA*, CDA*, CCDLSA* and CCDGA*.

Algorithm	1	2	3	4	5	6	7	8	9	10	Ave	Ratio
BFA*	1.787	1.711	1.620	1.712	1.738	1.883	1.702	1.782	1.738	1.680	1.74	100.00%
CDA*	1.644	1.756	1.711	1.668	1.676	1.640	1.687	1.650	1.682	1.64	1.68	96.55%
CCDLSA*	1.165	1.163	1.156	1.169	1.131	1.19	1.134	1.144	1.147	1.153	1.16	66.67%
CCDGA*	1.191	1.24	1.252	1.311	1.183	1.183	1.178	1.256	1.267	1.319	1.24	71.26%

**Table 6:** Tried Candidates, Expanded Nodes and Nodes in Queue for Single Diagnosis.

Algorithm	BFA*		CDA*		CCDLSA*		CCDGA*	
	Num	Ratio	Num	Ratio	Num	Ratio	Num	Ratio
Tried Candidates	11	100.00%	8	72.72%	3	27.27%	3	27.27%
Expanded Nodes	125	100.00%	116	92.80%	6	4.80%	23	18.40%
Nodes in Queue	293	100.00%	286	97.61%	15	5.12%	55	18.77%

**Table 7:** 3-Diagnosis Time(s) for BFA*, CDA* and CCDGA*.

Algorithm	1	2	3	4	5	6	7	8	9	10	Ave	Ratio
BFA*	2.326	2.234	2.228	2.179	2.146	2.152	2.060	2.227	2.122	2.163	2.18	100.00%
CDA*	1.844	1.951	2.053	1.823	1.881	1.871	1.815	1.871	1.890	1.892	1.89	86.70%
CCDGA*	1.347	1.593	1.465	1.642	1.534	1.517	1.477	1.496	1.482	1.524	1.52	69.72%

**Table 8:** Tried Candidates, Expanded Nodes and Nodes in Queue for Single Diagnosis.

Algorithm	BFA*		CDA*		CCDGA*	
	Num	Ratio	Num	Ratio	Num	Ratio
Tried Candidates	35	100.00%	14	40.00%	9	25.71%
Expanded Nodes	297	100.00%	234	78.79%	78	26.26%
Nodes in Queue	689	100.00%	610	88.53%	190	27.58%
